# Correlation of Internal Organ Weights with Body Weight and Body Height in Normal Adult Zambians: A Case Study of Ndola Teaching Hospital

**DOI:** 10.1155/2018/4687538

**Published:** 2018-04-18

**Authors:** Lumamba Mubbunu, Kasonde Bowa, Volodymyer Petrenko, Moono Silitongo

**Affiliations:** ^1^Department of Basic Science, Michael Chilufya Sata School of Medicine, Copperbelt University, P.O. Box 71191, Ndola, Zambia; ^2^Department of Pathology, Ndola Teaching Hospital, Postal Agency, Ndola, Zambia

## Abstract

The objective of the research was to study the correlation of internal organ weights with body weight and length in normal adult Zambians. The study involved 114 (83 males and 31 females) forensic autopsies from Ndola Teaching Hospital done over a period of 12 months. The cases included autopsies of unnatural deaths including road traffic accidents and homicide. Cases where information about age and origin of the person was not available were left out of the study. The age of the decedents ranged from 16 to 85 years. The data was analyzed by Pearson correlation coefficient to determine correlation. *P* values less than 0.05 were considered to be statistically significant. It was observed that the heart, liver, left kidney, right kidney, brain, and left lung were positively correlated to body weight, while only the brain and the left lung were positively correlated to the height in the male population. In the female population, the heart, liver, right kidney, brain, and right lung were positively correlated to the weight of the body, while only the right kidney was positively correlated to the height of the body.

## 1. Introduction

The weight of internal organs is important in forensic medicine and pathology, because the weight of internal organs is useful in determining whether the organ is normal or pathological. Many diseases have been shown to change the weight of internal organs [1, 2]. The change in the weight of an internal organ can be used in interpreting the opinion regarding the cause of death during an autopsy. Changes in the weight of an internal organ may be the only evidence to show that an organ is not normal. For example, the elevated weight of the heart may be the only evidence to myocardial hypertrophy that is often macroscopically and microscopically difficult to recognize [3]. In a similar vein, in a study by Greaves in 2000 [4], he reported that changes in kidney weight may also reflect renal toxicity, tubular hypertrophy, or chronic progressive nephropathy. In forensic pathology as well as in clinical medicine any deviation from the normal weight of an internal organ would be indicative of a pathology to that organ or a compensatory mechanism in response to applied stress to an organ. For example, if there is increased pulmonary resistance, the right ventricle of the heart may enlarge in response to the increase in the pressure required to pump blood through the pulmonary circulation [5]. The increase in the weight of an internal organ can only be determined if there is a reference weight, in this case the recorded weight of that internal organ to which the weight of an internal organ can be compared to. Since the determination of the normal weight of an internal organ is based on comparison with the reference data, the reference data should as much as possible be representative and be as accurate as can be to show that the reference weight shows the normal value [6]. The weight of internal organs can be a good diagnostic criteria for interpreting autopsy information if the weights of internal organs are compared to the appropriate reference weights [7, 8]. This means that normality for the weight of internal organs in a given population should be accurately defined. One of the ways to define this data is to generate reference tables for that population, meaning the average weight to be used as reference information should be generated from the people of that population. Another method that can be used to normalize the information is correlation of internal organ weights to body length and body weight. As it stands Zambian pathologists use references from American or European textbooks. The problem with these sources of information is that they may not be suitable for the Zambian population when interpreting postmortem cases. This could lead to inaccurate interpretation of postmortem cases. Human internal organ weights not only are determined by race, age, or gender but are also anticipated to be dependent on environmental and social economic conditions which may be quite different in various parts of the world. Hence, the weights of organs reported from other countries may not be the same as that for Zambian population. The current work was undertaken to study the average weight of Zambian population and correlate the weight of internal organs with body weight and height.

## 2. Materials and Methods

The study was undertaken at Ndola Teaching Hospital mortuary. The organs were weighed from selected bodies that satisfied the criteria for inclusion in the study. The bodies were selected from postmortem cases brought to the pathologists for forensic investigations for a period of 12 months. During this period, 114 cases were sampled, and all the autopsies that were used in the study were done by the same pathologist and anatomist. In the study, subjects who died due to natural causes were not included; this was because the organs in patients succumbing to a variety of morbid anatomical lesions or disease process were considered to be pathological. The assumption was that the ideal subjects for establishing the weight of internal organs would for those dying from accidental and violent deaths [9]. The assumption is that decedents who die accidentally are healthy. For the purposes of this study, normal organ was defined as an organ from a person who did not have any evidence of any type of disease or infection that would damage that organ: the gross appearance of the organs had to be without any evidence of any type of damage and or pathology.

### 2.1. Inclusion Criteria

Cases of accidents and homicide, where there was no evidence of gross pathology or trauma to the internal organs of interest were selected for inclusion. Postmortem examination was carried out within 24 hours and petrification had not occurred on the body and organs. If the body stayed too long more than 48 hours in the fridges at the mortuary that case was not included. The decedents were all natives of Zambia. The autopsy case was a forensic or medical legal autopsy.

### 2.2. Exclusion Criteria

Cases not satisfying the above criteria were excluded.

### 2.3. Ethical Approval

*Ethical clearance* was granted by Tropical Diseases Research Center ethical review committee: IRB registration number: 00002911. FWA number is 00003729. The study number for this study is TDRC/ERC/01/07/2014.

## 3. Data Collection

The height was measured from head to heel using a height measure in centimeters. The body was weighed naked [6, 7]. Using a scale 0.0 Kg to 300 kg with accuracy of ±100 g, the scale was calibrated by the Zambia weight and measure agency and 0.5, 1.0, and 5.0 Kg control weights were used to calibrate the scale every time before it was used. The internal organs were weighed using a calibrated electronic balance 0.0 g to 5000 g with accuracy of ±0.1 g. The collected data was analyzed using IBM-SPSS program version 23. They were divided into gender: male and female. Person's correlation coefficient was used to analyze the relationships between body weight and body length of the internal organs at *P* < 0.05.

*Results. *The subjects of the study were divided into groups comprised of males and females.

## 4. Discussion

The sample population for this study was made up of people who had died in road traffic accidents and homicide cases that were brought to Ndola Teaching Hospital for autopsy. 114 cases were selected for the study: 31 females and 83 males. The age group for the females was 18–85 years with the average age of 39.9 ± 15.2 years, while that of the males was 16–85 years with an average of 38.1 ± 16.0 years (see Table 1). The weight and height of the decedents were also measured, and the average weight and height for the female population were 57.5 ± 11.2 Kg and 163.4 ± 6.7 cm, respectively, while those of the male population were 58.5 ± 11.2 Kg and 169.2 ± 7.84 cm (See Table 1). 90% of the samples decedents deaths were road traffic accidents and 10% suspected murder cases.

### 4.1. Heart

The weight of the heart was 279.2 ± 44.9 g in males and 268.4 ± 64.2 g in the female population representing 0.48% and 0.47% of body weight, respectively (Tables 1 and 3). Body weight percent of the heart was different when compared to other authors [10, 11]. The weight of the heart was positively correlated with body weight in both males and females at *P* < 0.05 (see Table 2). The weight of the heart was not correlated to the height of the body in both males and females (see Table 2). Other researchers [7] found that the weight of the heart was positively correlated to the weight in both males and females, while the weight of the heart was only positively correlated to the height of the body in males and not in female decedents in Thai population which is different from the findings of this study. In the same vein, Hanzlick and Rydzewski [12] found that the weight of the heart in males is correlated to body weight and length, whereas the weight of the heart in females is correlated only to body weight but not correlated to body height.

### 4.2. Spleen

The weight of the spleen was found to be 169.9 ± 107.2 g in males and 161.7±93.4 g in females (Table 1). In this study, the weight of the spleen was not correlated to the weight and height of the spleen at *P* < 0.05 (Table 2). Other authors have indicated that the spleen is positively correlated to body weight in males [7]. Similarly, Mathuramon et al. [6] found that the weight of the spleen is positively correlated to body weight and height in males and not in females. It is postulated that this may have been because the population that was studied is in a malaria endemic country and the size of the spleen could have been due to parasitic infections. Parasitic infections have been observed to cause increase to the weight of the spleen [13]. The weight of the spleen was 0.28% in males and 0.28% in females (Table 3). The figures in this study are higher than the findings of Tanna et al. [14] in which they found percent body weight of the spleen at 0.15–0.25% in males and 0.18–0.24% in females.

### 4.3. Liver

The weight of the liver was found to be 1285.3 ± 270.1 g and that of females 1367.9 ± 357.2 g (Table 1). The weight of the liver was positively correlated to body weight in both males and females at *P* < 0.05 (Table 2). The finding is similar to the findings in the Thai population [6]; however the weight of the liver did not correlate with the height of the decedents in the study (see Table 2). The percent body weight of the liver in males was 2.2% while it was 2.4% in female decedents (see Table 3). The findings are different from those of Standring [10] and Tanna et al. [14].

### 4.4. Lungs

The weight of the lungs was found to be 442.0 ± 152.3 g left lung and 504.6 ± 174.6 g right lung in the male population while in the female population the weight of the left lung was 365.7 ± 119.8 and the right lung 405.0 ± 116.0 g (Table 1). The percent body weight of the lungs in males was 0.76% for the left lung and 0.86% for the right lung. In the female decedents, the left lung was 0.64% and the right lung was 0.70% of body weight (Table 3). The findings in this study are different from other researchers [14]. The weight of the left lung was positively correlated to the weight and height of the body in males; however there was no correlation with either body weight or body height in the female decedents at *P* < 0.05 (Table 2). The weight of the right lung was positively correlated to the weight of the body in females while there was no correlation with the height of the decedents. In the male decedents, there was no correlation with the weight of the right lung with body weight and body height. Other studies which have shown that there is a positive correlation between body weights in males had the weight of the lungs combined to give total lung weight [6, 7, 15]. In this work, the left lung and right lung were analyzed independent of each other.

### 4.5. Kidneys

The weight of the kidneys was found to be 110.0 ± 22.8 g right kidney and 117.9 ± 27.4 left kidney in the males and 101.2 ± 20.3 g right kidney and 108.3 ± 23.0 g left kidney in females (Table 1). The weight of the left kidney and right kidney represented 0.20% and 0.19%, respectively, in the male decedents (see Table 3). The left kidney represented 0.19% and the right kidney 0.18% in females (Table 1). The findings are similar to findings by Tanna et al. [14] for the percentage body weight of the kidneys. Both the left and right kidney were positively correlated to body weight at *P* < 0.05 (Table 2), while there was no correlation for both the left and right kidney with body height in the male decedents. In the female decedents, the right kidney was positively correlated with body weight and height while the left kidney was not. This is different from the findings of Prakash et al. 2013. Other authors have shown that the combined weight of the kidneys is positively correlated to body weight and height in both male and female decedents [6].

### 4.6. Brain

The weight of the brain was 1335.0 ± 25.5 g in the male decedents and 1228.3 ± 76.4 g in females representing 2.28% and 2.31% of body weight, respectively, in male and female decedents (see Tables 1 and 3). The average weight of the brain in this study was positively correlated with the weight and height of the male decedents, while, in female decedents, the weight of the brain was positively correlated with the weight of the body but not the height of the body (Table 2). The percentage body weight of the liver in this population was greater than that of the Bhavnagar region of India [14]. A study by Mathuramon et al. [6] shows that the weight of the brain is not positively correlated to the height of the body in the decedents in both males and females; this is different from the findings of this study. Prakash et al. [15] however found that the weight of the brain was positively correlated to the weight of the body in female and not in male decedents.

### 4.7. Strengths of the Study

It should be noted that this study differs from other studies because the data was collected prospectively. By so doing the researchers were able to see the subjects which gave the researchers more information on the rejection and inclusion criteria, other studies that have used retrospective records to analyze the correlation of internal organ weights with body weight and height relied on information collected by others and may include organ weights from subjects that maybe otherwise would have been rejected. The same pathologist and anatomist collected the data which means that the method of cutting and weighing the organs was consistent thereby reducing errors which would arise from having many people involved in collection of data.

### 4.8. Limitation and Weaknesses of the Study

The study had the following limitations: there was no information about the kind of life the subjects in the study lived and the medical condition of the subject at the time of death was not known. The answers about the medical condition of the subjects would have helped a great deal in refining the weight of internal organs. Histological studies could have also helped in removing weights of internal organs that may have looked normal or were of gross appearance and yet pathological. It was not easy to get a large sample size, especially with the female sample which were 32 in number, in which case the interpretation of the data for the female population should be done with a lot of caution. The study may not be suitable in interpreting autopsy of people from other provinces within Zambia.

## 5. Conclusion

In this study, it was observed that the heart, liver, left kidney, right kidney, brain, and left lung were positively correlated to body weight, while only the brain and the left lung were positively correlated to the height in the male population. In the female population, the heart, liver, right kidney, brain, and right lung were positively correlated to the weight of the body, while only the right kidney was positively correlated to the height of the body. Further work is required with a larger sample population to ascertain the true relationships of the weight of internal organ with body weight and height.

## 6. Recommendations

Further work will need to be done in this area with a large population of at least 2000 subjects to improve the statistical validity of the arguments. All regions of Zambia will have to be covered not just one center like Ndola central hospital in this case. Multiple centers and increased period of study may increase the sample size.

## Figures and Tables

**Table 1:** Mean, standard deviation, maximum of body weight, body height, age, and weight of internal organs.

	Male *N* = 83		Female *N* = 31	
	Min–max	Mean and SD	Min–max	Mean and SD
Age	16–85 years	38.1 ± 16.0 years	18–85 years	39.9 ± 15.2 years
Weight	33 Kg–87 Kg	58.5 ± 11.20 Kg	36–77 Kg	57.5 ± 11.2 Kg
Height	149–187 cm	169.0 ± 7.84 cm	151–176 cm	163.4 ± 6.7 cm
Heart	169–379 g	279.2 ± 44.9 g	180–451 g	268.4 ± 64.2 g
Liver	649–1894 g	1285.3 ± 270.1 g	828–2154 g	1367.9 ± 357.2 g
Spleen	44–468 g	169.9 ± 107.2 g	28–515 g	161.7 ± 93.4 g
Right kidney	70–175 g	110.0 ± 22.8 g	61–152 g	101.2 ± 20.3 g
Left kidney	71–193 g	117.9 ± 27.4 g	75–180 g	108.3 ± 23.0 g
Brain	1054–1630	1335.0 g ± 125.5	1064–1400 g	1228.3 ± 76.4 g
Left lung	144–922	442.0 g ± 152.3 g	216–691 g	365.7 ± 119.8 g
Right lung	230–1053	504.6 ± 174.6 g	235–795 g	405.0 ± 116.0 g

**Table 2:** Correlation between internal organ weight and body weight and height.

Organ	Male *N* = 83				Female *N* = 31			
	Body weight		Body height		Body weight		Body height	
Heart	*r* = 0.575	*P* < 0.001	*r* = 0.211	*P* = 0.056	*r* = 0.353	*P* = 0.047	*r* = 0.349	*P* = 0.050
Liver	*r* = 0.310	*P* = 0.004	*r* = 0.045	*P* = 0.685	*r* = 0.554	*P* < 0.001	*r* = 0.291	*P* = 0.106
Spleen	*r* = 0.001	*P* = 0.996	*r* = −0.102	*P* = 0.361	*r* = 0.198	*P* = 0.227	*r* = 0.295	*P* = 0.102
Left kidney	*r* = 0.331	*P* = 0.002	*r* = 0.018	*P* = 0.875	*r* = 0.256	*P* = 0.157	*r* = 0.236	*P* = 0.193
Right kidney	*r* = 0.275	*P* = 0.013	*r* = −0.013	*P* = 0.906	*r* = 0.400	*P* = 0.023	*r* = 0.368	*P* = 0.038
Brain	*r* = 0.490	*P* < 0.001	*r* = 0.401	*P* < 0.001	*r* = 0.396	*P* = 0.025	*r* = −0.049	*P* = 0.788
Left lung	*r* = 0.259	*P* = 0.018	*r* = 0.237	*P* = 0.031	*r* = 0.242	*P* = 0.182	*r* = 0.128	*P* = 0.483
Right lung	*r* = 0.130	*P* = 0.168	*r* = 0.196	*P* = 0.076	*r* = 0.394	*P* = 0.026	*r* = 0.096	*P* = 0.600

The percent body weight was also calculated for the weight of internal organs. This was done to get an estimate of percent body weight for each organ that was studied.

**Table 3:** Percent body weight of the organs in both male and female population.

Organ	Weight	Male % Body weight	Organ	Weight	Female % Body weight
Heart	279.2 ± 44.9 g	0.48%	Heart	268.4 ± 64.2 g	0.47%
Liver	1285.3 ± 270.1 g	2.20%	Liver	1367.9 ± 357.2 g	2.38%
Spleen	169.9 ± 107.2 g	0.29%	Spleen	161.7 ± 93.4 g	0.28%
Left kidney	117.9 ± 27.4 g	0.20%	Left kidney	108.3 ± 23.0 g	0.19%
Right kidney	110.0 ± 22.8 g	0.19%	Right kidney	101.2 ± 20.3 g	0.18%
Brain	1335.0 g ± 125.5	2.28%	Brain	1228.3 ± 76.4 g	2.13%
Left lung	442.0 g ± 152.3 g	0.76%	Left lung	365.7 ± 119.8 g	0.64%
Right lung	504.6 ± 174.6 g	0.86%	Right lung	405.0 ± 116.0 g	0.70%
Body weight	58.5 ± 11.2 Kg		Body weight	57.47 ± 11.2 Kg	
